# Whole-Genome Sequences of 26 *Vibrio cholerae* Isolates

**DOI:** 10.1128/genomeA.01396-16

**Published:** 2016-12-22

**Authors:** Samit S. Watve, Aroon T. Chande, Lavanya Rishishwar, Leonardo Mariño-Ramírez, I. King Jordan, Brian K. Hammer

**Affiliations:** a School of the Biological Sciences, Georgia Institute of Technology, Atlanta, Georgia, USA; b Applied Bioinformatics Laboratory, Atlanta, Georgia, USA; c PanAmerican Bioinformatics Institute, Cali, Valle del Cauca, Colombia; d National Center for Biotechnology Information, National Institutes of Health, Bethesda, Maryland, USA

## Abstract

The human pathogen *Vibrio cholerae* employs several adaptive mechanisms for environmental persistence, including natural transformation and type VI secretion, creating a reservoir for the spread of disease. Here, we report whole-genome sequences of 26 diverse *V. cholerae* isolates, significantly increasing the sequence diversity of publicly available *V. cholerae* genomes.

## GENOME ANNOUNCEMENT

*Vibrio cholerae* is a Gram-negative, facultative anaerobe commonly found in salt water and can cause the fatal diarrheal disease cholera. *V. cholerae* is spread predominantly by fecal contamination of water and food sources in both endemic and epidemic regions (1). Governmental and nongovernmental labs worldwide collect *V. cholerae* isolates for standard bacterial surveillance, and many clinical isolates of *V. cholerae* have already been sequenced (2–4). Clinical samples are often isolated from limited geographic regions and clonally derived with few or no genetic differences (5–8). An expanded characterization of genomes from environmental isolates of *V. cholerae*, which tend to be far more genetically and phenotypically diverse (9), should substantially increase the available sequence diversity of this important human pathogen.

*Vibrio* spp. are known to encode type VI secretion systems (T6SS), which are often described as bacterial weapons designed to pierce the membranes of adjacent cells and deliver toxic effectors that can lead to lysis of target (prey) cells. In a recent survey, Bernardy et al. (10) noted key differences within a diverse set of isolates for several phenotypes, including chitinase production, contact-dependent killing indicative of T6SS activity, and natural transformation, which can promote horizontal gene transfer. Both clinical and environmental isolates were rarely naturally transformable. In contrast, the majority of environmental, but not clinical, isolates constitutively killed *Escherichia coli* prey. Because different regulatory schemes control the phenotypes tested (11, 12), we sought to better understand the genetics that underlie these diverse *V. cholerae* phenotypes by characterizing whole-genome sequences of 23 environmental and three clinical isolates from Bernardy et al.

All strains were grown overnight in LB medium (Difco) at 37°C, with shaking. Genomic DNA was isolated using a ZR fungal/bacterial DNA mini prep kit (Zymo Research), and paired-end fragment libraries were constructed using a Nextera XT DNA library preparation kit (Illumina) with a fragment length of 300 bp. Libraries were sequenced by the High Throughput Sequencing Core at Georgia Institute of Technology on an Illumina HiSeq 2500 Rapid platform, producing approximately 280 million 100-bp reads in total. Reads were trimmed using Trimmomatic (13) to remove adapters and bases with a read quality score of <20. Genomes were assembled using SPAdes version 3.5 (14) and annotated using the Rapid Annotation and Subsystem Technology (RAST) web tool provided by the National Microbial Pathogen Data Resource (15–18). T6SS genes were annotated using T6SS Predictor (A. T. Chande et al., unpublished data).

T6SS loci were annotated in all genomes in an effort to characterize the genetic basis of T6SS-mediated killing among diverse environmental *V. cholerae* isolates. All genomes were found to encode the previously characterized large cluster and two auxiliary clusters, which together comprise the canonical T6SS loci. In addition, two previously unreported T6SS loci were discovered in six of the isolates. Numerous examples of novel effector-immunity protein pairs, which function together to catalyze T6SS-mediated killing, were characterized among the set of environmental isolate genomes. Taken together, our genome analysis illuminates the diverse repertoire of genetic mechanisms that underlie T6SS-mediated killing in *V. cholerae*.

### Accession number(s).

This whole-genome shotgun project has been deposited at DDBJ/EMBL/GenBank under the accession numbers listed in Table 1.

**TABLE 1:** List of *V. cholerae* strains sequenced in this study

Strain^a^	Location	Source	Yr of isolation	Type VI killing activity^b^	NCBI accession no.
1496-86	United States (LA)	Moore swab	1986	−	MIPC00000000
2523-87	United States (LA)	Moore swab	1974	+	MIPE00000000
VC48	United States (FL)	Oyster	1981	+	MIOT00000000
2633-78	Brazil	Sewage	1978	+	MIPH00000000
857	Bangladesh	Water	1996	+	MIKH00000000
3272-78	United States (MD)	Water	1977	+	MIOZ00000000
TP	United States (CA)	Water	2000	+	MIPK00000000
2559-78	United States (LA)	Crab	1978	+	MIOW00000000
HE46	Haiti (center)	Gray water	2011	+	MIPM00000000
2479-86	United States (LA)	Moore swab	1986	+	MIPB00000000
2497-86	United States (LA)	Moore swab	1987	+	MIPD00000000
2512-86	United States (LA)	Moore swab	1986	+	MIOY00000000
2631-78	United States (LA)	Moore swab	1978	+	MIOX00000000
VC22	United States (FL)	Oyster	1981	+	MIKK00000000
VC53	United States (AL)	Oyster	2009	+	MIOU00000000
VC56	United States (AL)	Oyster	2009	+	MIOV00000000
3568-07	Mexico	Queso fresco	2007	+	MIPL00000000
1074-78	Brazil	Sewage	1978	+	MIPG00000000
3223-74	Guam	Storm drain	1974	+	MIZG00000000
3225-74	Guam	Storm drain	1974	+	MIPF00000000
2740-80	United States (Gulf Coast)	Water	1980	+	MIKI00000000
692-79	United States (LA)	Water	1979	+	MIPA00000000
SIO	United States (CA)	Water	2000	+	MIPJ00000000
C6706	Peru	Patient	1991	−	MIPI00000000
MZO-2	Bangladesh	Patient	2001	−	MIKJ00000000
V52	Sudan	Patient	1968	+	MIPN00000000

^a^ Strains were isolated from an environmental source, except strains C6706, MZO-2, and V52.

^b^ Presence (+) or absence (−) of constitutive type VI killing activity.
